# Overlap of generalized morphea and eosinophilic fasciitis after recreational exposure to epoxy resin

**DOI:** 10.1016/j.jdcr.2025.01.012

**Published:** 2025-02-04

**Authors:** Noah Hewitt, Kelly Tran, Josiah Williams, Patrick Bacaj, Erika Poling, Alicia Zbehlik

**Affiliations:** a Department of Medicine, West Virginia University School of Medicine, Morgantown, West Virginia; b Department of Dermatology, West Virginia University School of Medicine, Morgantown, West Virginia; c Department of Pathology, Anatomy, and Laboratory Medicine, West Virginia University School of Medicine, Morgantown, West Virginia; d Division of Rheumatology, Department of Medicine, West Virginia University School of Medicine, Morgantown, West Virginia

**Keywords:** dermatopathology, eosinophilic fasciitis, eosinophils, epoxy resin, morphea, scleroderma

## Introduction

Morphea, or localized scleroderma, is a connective tissue disorder causing inflammation and sclerosis of skin and underlying tissues.^1^ Clinical presentation exists on a spectrum with varying degrees of severity and anatomical involvement.^2^ Eosinophilic fasciitis (EF) is often considered a severe, deep form of morphea that may result in significant morbidity.^2^ Although idiopathic in most cases, morphea may develop after certain environmental and occupational exposures.^1^^,^^3^ The development of a morphea-like reaction after exposure to epoxy resin is a rare phenomenon reported in the literature.^4^ We present a case of generalized morphea and EF overlap presenting in a middle-aged woman after recreational exposure to epoxy resin.

## Case report

A 44-year-old woman with unremarkable medical history presented to rheumatology clinic for stiffening of her skin. In 2021, she noticed hyperpigmented, pruritic patches over her trunk and lower extremities following a spill of epoxy resin on her thighs while using the compound for creating decorations. For 2 years, this remained relatively stable, but she had 2 additional epoxy resin spills in 2022 and 2023. Six months prior to presentation, the plaques rapidly expanded and became confluent on all extremities. This led to upper and lower extremity weakness, skin tightness, and limited range of motion of her fingers with inability to form a fist or grip a steering wheel. Workup prior to presentation included electromyography demonstrating carpal tunnel syndrome.

**Fig 1:**
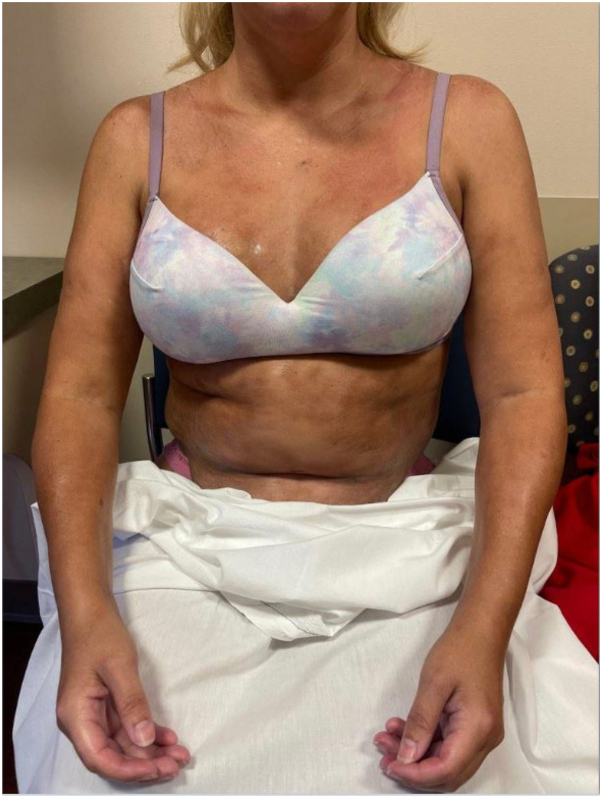
Initial clinical presentation demonstrating several hyperpigmented, depressed plaques on her trunk and confluent indurated plaques on her upper extremities. The extent of finger flexion bilaterally can also be appreciated.

**Fig 2:**
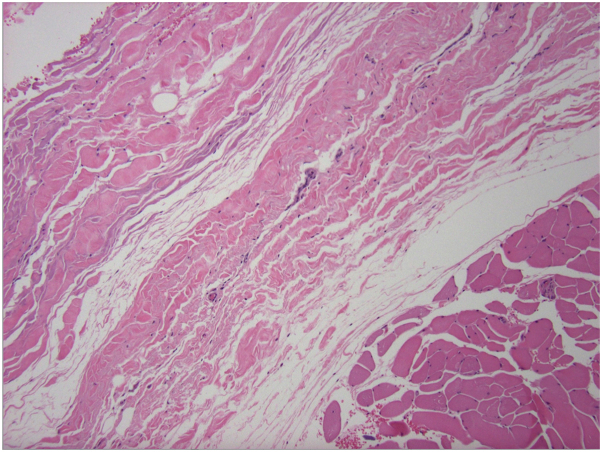
Microscopic findings demonstrating thickened subcutaneous fascia with adjacent skeletal muscle. H&E 100× magnification.

**Fig 3:**
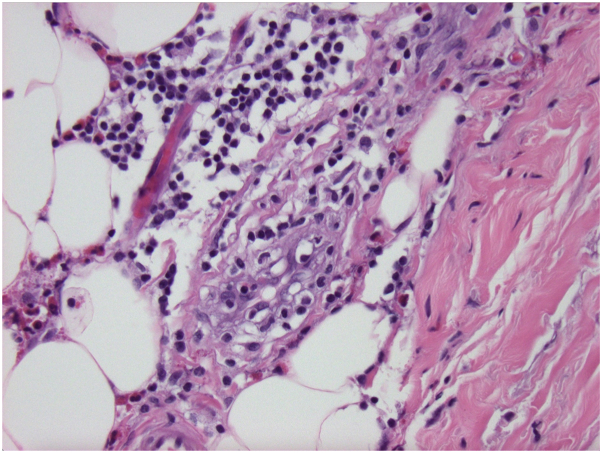
Microscopic findings demonstrating deep perivascular lymphoeosinophilic inflammation. H&E 400× magnification.

Upon exam, she had several hyperpigmented, depressed plaques on her trunk and confluent indurated plaques on her upper extremities (Fig 1). Identical skin lesions were also present on her lower extremities. She had synovitis with tenderness to palpation of her metacarpal phalangeal joints and wrists. She also had limitation of finger flexion bilaterally. The extent of her ability to flex her fingers can be appreciated in Fig 1. On dermoscopy, there were no dilated nailbed capillaries. Laboratory evaluation was notable for absolute eosinophil count of 1530 cells/uL and elevated C-reactive protein (31 mg/L). Rheumatology workup was otherwise unremarkable, including negative results for antinuclear antibodies, anticentromere antibodies, and antitopoisomerase I antibodies. The patient was referred to dermatology and initial punch biopsy revealed increased dermal collagen with perivascular lymphoeosinophilic infiltrate but was not deep enough to evaluate fascia. Follow-up incisional biopsy demonstrated dermal sclerosis, thickened fascia (Fig 2), and deep perivascular lymphoeosinophilic inflammation (Fig 3). The patient was initiated on treatment as highlighted in Table I. Although she was initially unable to schedule physical therapy, she is currently under their care with gradual improvement.

**Table I:** Timeline of treatment, laboratory values, patient-reported response, and clinical response

Date	11/2023 (Baseline)	12/2023	1/2024	2/2024	3/2024	5/2024	6/2024	10/2024
Prednisone PO		40 mg daily	30 mg daily	25 mg daily	15 mg daily	5 mg daily	5 mg daily	5 mg daily
MTX PO		10 mg weekly	15 mg weekly	20 mg weekly	20 mg weekly	20 mg weekly	20 mg weekly	20 mg weekly
MMF PO						0.5 g daily	1 g daily	1.5 g daily
Eosinophils cells/uL (x 1000)	1.53	<0.10		<0.10	<0.10	<0.10	0.20	<0.10
CRP mg/L	31.0	0.9		0.7	3.5	3.7	27.7	4.5
ESR mm/hr	47	23		8	10	14	16	23
Patient comments	Unable to grip a steering wheel	Decreased swelling, 20% improvement		Feels 100% better, some increase in pain with decrease in prednisone dosage		Notes skin tightening, decreased pain, improved mobility	Increased dexterity, able to braid her hair and interlock fingers for the first time in years	Continued improvement in dexterity
Physical exam	Synovitis, hands and wrists, skin edematous and thickened with diffuse hypo- and hyperpigmented patches on trunk and extremities	Slightly improved synovitis, unable to make a fist (see hands in Fig 1)		Decreased synovitis, unable to make a fist, walking with a walker		Decreased synovitis, unable to make a fist		Mild synovitis at wrists, unable to make fist with 5 cm from tip of digits to palmar crease

*CRP*, C-reactive protein; *ESR*, erythrocyte sedimentation rate; *MMF*, mycophenolate mofetil; *MTX*, methotrexate; *PO*, by mouth.

## Discussion

Eosinophilic fasciitis is often considered a severe form of morphea that causes inflammation and sclerosis of muscle fascia with unclear pathogenesis.^1^^,^^2^^,^^5^ It mostly affects middle-aged adults in their fourth or fifth decade of life.^2^ Concomitant conventional morphea is present in 29% to 40% of cases.^1^^,^^2^^,^^5^ Although EF is mostly considered idiopathic, various potential triggers and associations have been identified including strenuous exercise, autoimmune diseases, medications, hematologic disorders, cancer, hemodialysis, Lyme disease, and graft-versus-host disease, and ingestion of tryptophan.^5^^,^^6^

Epoxy resin exposure is also associated with sclerotic skin disorders.^3^^,^^4^^,^^7^ Although the exact mechanism is not fully understood, several possible mechanisms have been proposed. Bis(4-amino-3-methylcyclohexyl)methane, an amine present in epoxy fumes, is one possible culprit in initiating the sclerosing process; likewise, epoxy resin oligomers may start the process by binding to amines found in tissues.^4^^,^^7^ A strong allergic reaction to epoxy resin oligomers has also been postulated as a cause for sclerosis.^3^^,^^4^^,^^7^ Although causality is uncertain, the temporal association between epoxy resin spills and the location of skin contact suggest it could have contributed to this patient’s presentation.

Eosinophilic fasciitis typically presents with circumferential non-pitting edema, eventually progressing to induration with an “orange peel” appearance.^2^^,^^5^ Early stages of disease may present with localized pain and erythema.^5^ Progression to induration indicates the development of sclerosis in deeper tissues.^2^ The “groove sign” may be present, which refers to the development of a linear depression seen on the surface of the skin as underlying vessels traverse through areas of sclerosis.^5^ Patients may endorse weight loss, myalgia, weakness, and fatigue.^2^ Inflammatory arthritis and limitations in joint mobility may be present due to significant fascial fibrosis.^5^ Some patients may develop neurologic manifestations such as carpal tunnel syndrome.^5^ Visceral involvement is uncommon.^5^

Laboratory tests usually demonstrate transient peripheral eosinophilia, elevated acute phase reactants such as C-reactive protein, and hypergammaglobulinemia.^5^ A full-thickness biopsy of deep muscular fascia is the gold standard for establishing the diagnosis.^2^^,^^5^ Positive biopsies demonstrate fascial fibrosis with infiltration of lymphocytes, eosinophils, plasma cells, and monocytes.^2^^,^^5^ Tissue eosinophilia may be transient, making diagnosis challenging.^2^^,^^5^ In patients with chronic disease or those who have received immunosuppressive medications, tissue eosinophilia may be absent.^2^ In these instances, magnetic resonance imaging can help identify fascial inflammation.^2^^,^^5^ Due to similarities in clinical presentation, differentiating between deep morphea and EF may be challenging, although a true distinction may not exist since many consider these diseases to exist on a spectrum.^2^ In general, a symmetrical distribution, a prominent inflammatory phase, and peripheral eosinophilia favor the diagnosis of EF. Diagnostic criteria for eosinophilic fasciitis have been proposed. The major criterion requires patients to present with symmetrical plate-like sclerotic lesions on the 4 limbs characteristic of EF.^8^ One of 2 minor criteria must be present, which include characteristic biopsy findings or a magnetic resonance imaging demonstrating fascial thickening; extensive treatment prior to these studies could affect their sensitivity.^8^

Initial treatment includes high-dose systemic corticosteroids, typically starting at prednisone 1 mg/kg/day.^5^ Patients who remain unresponsive to high-dose systemic corticosteroids without resolution of peripheral eosinophilia, improved inflammatory markers, or skin softening may be transitioned to methotrexate 15-20 mg weekly.^5^ Other treatment options include mycophenolate mofetil or hydroxychloroquine, although data on their efficacy are limited.^5^ In refractory cases, tocilizumab, baricitinib, sulfasalazine, azathioprine, infliximab, rituximab, intravenous immunoglobulins, dapsone, ultraviolet A 1 phototherapy, psoralen plus photochemotherapy UVA, cyclosporine A, and sirolimus have been trialed with varying results.^5^ Patients with significant functional limitation may benefit from physical therapy and surgery, such as fasciotomy.^5^ Reslizumab and mepolizumab are new anti-IL-5 infusion-based monoclonal antibody agents that may be promising treatment options for refractory cases.^5^

## Conflicts of interest

None disclosed.
